# Evaluation of Delayed Effective Discharge for Non-Medical Reasons in Patients Admitted to Acute Care Hospitals in Spain: A Scoping Review

**DOI:** 10.3390/nursrep14010002

**Published:** 2023-12-23

**Authors:** Noelia López Luis, Cristobalina Rodríguez-Álvarez, Leticia Cuéllar-Pompa, Ángeles Arias

**Affiliations:** 1 Primary Care Management of Tenerife, Canary Islands Health Service, 38004 Santa Cruz de Tenerife, Spain; alu0100754629@ull.edu.es; 2 Department of Preventive Medicine and Public Health, University of La Laguna, 38320 Santa Cruz de Tenerife, Spain; crrodrig@ull.edu.es; 3 Institute of Care Research of the Nurses Association of Santa Cruz de Tenerife, 38001 Santa Cruz de Tenerife, Spain; alu0100571547@ull.edu.es

**Keywords:** patient discharge, length of stay, transfer agreement, review

## Abstract

Delayed discharge for non-clinical reasons is defined as a period of stay that continues after a patient has been deemed medically fit to leave the hospital but is unable to do so for non-medical reasons. This circumstance overburdens the healthcare system and constitutes a major problem for healthcare systems and the patients themselves in this situation. The aim of this study was to evaluate the delay in effective discharge for non-medical reasons for patients admitted to acute care hospitals in Spain. A scoping review followed the Joanna Briggs Institute methodology guidelines to search for and synthesize studies published between 2019 and 2022. To identify potentially relevant documents, the following bibliographic databases were searched: EMBASE, CINAHL and MEDLINE. For the search, we used free terms («delayed discharges», «discharge delays», «bed-blocking», «timely discharge», «unnecessary days» and «inappropriate stays»). Quantitative or qualitative studies published in scientific journals on delayed effective discharge for non-medical reasons for patients admitted to a hospital for any health issue of medical or surgical origin were selected. Information collection of the documents was performed using a structured datasheet specifically developed by the authors. The initial search strategy identified a total of 124 references, which were successively screened to a final selection of 13 studies. To conclude, delayed discharge from a hospital for non-clinical reasons is a multifactorial problem. This may be due to factors internal or external to the hospital, as well as personal factors. The main causes of the delay were similar among the studies found, as were the clinical characteristics of the patients, most of whom were elderly, frail and more dependent due to declining functional capacities. Further studies addressing the socio-familial characteristics of the patients and the perspective of the patient and families would be necessary.

## 1. Introduction

Delayed hospital discharge is a quality-of-care issue shared by many healthcare systems worldwide [1]. It is defined as “a period of stay that continues after a patient has been deemed medically fit to leave the hospital but is unable to do so for non-medical reasons”. An increasing proportion of patients experiences a delay in hospital discharge after it is deemed medically appropriate to be discharged from the hospital [2].

This circumstance overburdens the healthcare system and constitutes a major problem for healthcare systems and the patients themselves in this situation, as care normally diminishes or stops completely while they are waiting to be discharged, putting patients at risk of functional impairment [3], falls [4] and infections [5]. Previous reviews found a link between delayed discharge and morbidity and mortality in older people. Delayed discharges are not simply a product of increasingly complex patients’ needs but the result of inefficiencies within and between health sectors and organizations and a failure of health policy [6].

Progressive aging of the population and an increased number of dependent people, together with social changes experienced in the last few decades, have modified socio-familial structures. According to projections from the National Institute of Statistics (INE by its acronym in Spanish), people aged 65 and over will account for 26.5% of the whole Spanish population by 2035 [7]. Moreover, a high prevalence of loneliness among the elderly has been proven to be linked to health and satisfaction with formal and informal care [8]. According to these data, delayed discharges are expected to be an emerging issue in Spain. However, the incidence of this problem and its clinical and socio-familial characteristics have been scarcely evaluated to date in the country.

Spain has a national health service that provides tax-funded universal care, with each autonomous community being responsible for managing their own health services. In 2006, the «Law to promote personal autonomy and care for dependent people» was passed, a project to finance the needs of dependent people [9].

There was also found to be a link between high dependency and delayed discharges, with social isolation playing a major role [6]. Delayed discharges are known to be strongly affected by the level of coordination between health and social care systems in the long term. Thus, the ongoing expansion of care to dependent people since the passing of the law has been proven to help reduce inefficiencies in hospital care, as well as the number and frequency of hospitalizations [8].

A study carried out in Spain in 2009 on patients admitted to internal medicine (IM) units found a 3.5% incidence of discharge delays, mainly motivated by socio-familial difficulties in caring for patients after hospital admission [10]. Another study performed between 2007 and 2015 studying all cases of delayed discharges in the hospitalization units of the General University Hospital of Cantabria identified 3015 cases of delayed discharges during the study period. This amounts to a proportion of 0.93% of all discharges [11]. Both studies share the limitations of identifying the characteristics of the patient and his or her environment and functional dependency, which can lead to endogeneity when explaining discharge delays.

Furthermore, the issue of delayed discharges is influenced by local social and health resources, thus limiting the results and their comparability. The findings can help identify the necessary realistic measures to improve this situation in Spain.

In light of the above, the study of delayed discharge for non-clinical reasons is relevant mainly because demographic evolution can worsen the issue. This study aims at analyzing delayed effective discharges for non-medical reasons for patients admitted to acute care hospitals in Spain.

## 2. Materials and Methods

A scoping review was carried out in order to answer our research question: What are the consequences of delayed effective discharge for non-medical reasons for patients admitted to acute care hospitals in Spain?

It was performed following the Arksey and O’Malley protocol [12], Preferred Reporting Items for Systematic Reviews and Meta-Analyses (PRISMA) criteria for scoping reviews [13] and the Joanna Briggs Institute (JBI) manual [14]. This study was prospectively registered with the Open Science Framework (OSF) with registration number DOI 10.17605/OSF.IO/HA9G5.

### 2.1. Search Strategy

The review was carried out according to a five-step methodological framework, which was used for additional support following the Arksey and O’Malley criteria [12]:

Stage 1: Identification of the research question.

Stage 2: Identification of pertinent or relevant studies.

Stage 3: Study selection according to inclusion criteria.

Stage 4: Data registration and reporting.

Stage 5: Collection, summary and communication of results.

Firstly, a search was carried out to contextualize and update the research topic using databases such as PubMed and resources like ScienceDirect. Aside from allowing to us analyse the current scenario of the research topic, this also permitted identifying the descriptors («delayed discharges», «discharge delays», «bed-blocking», «timely discharge», «unnecessary days» and «inappropriate stays») used later for searching the literature. In our search, we used a free term vocabulary because we considered that when searching through MeSH terms, only articles indexed in Medline were retrieved, while when using free vocabulary, we could search all PubMed records.

To identify potentially relevant documents, the following bibliographic databases were searched: EMBASE, CINAHL and MEDLINE. The search strategies were drafted by an experienced librarian and further refined through team discussion.

Additionally, we used a “snowball” strategy by consulting the references of the included studies.

Table 1 shows the search strategy applied on 28 March 2023 for each database used in the search process.

### 2.2. Study Selection Criteria

#### 2.2.1. Inclusion Criteria

Quantitative or qualitative studies published in scientific journals on delayed effective discharge for non-medical reasons for patients admitted to a hospital for any health issue of medical or surgical origin were selected.

Only studies published in English or Spanish were considered. The search period was from January to March 2023.

Studies analysing the impact and experiences of delayed discharge were included if they fulfilled one or more of the following inclusion criteria:

- Quantitative data on the impact of delayed discharge on health outcomes (e.g., care quality, satisfaction, number of infections, mental health, mortality, morbidity, readmissions and functionality);
- Qualitative data on delayed discharge experiences from the perspective of patients (e.g., perceived impact on health or patient’s experience), health professionals and hospitals;
- Information on delayed discharge costs due to unnecessary hospitalization days.

#### 2.2.2. Exclusion Criteria

Summaries, editorials, comments and book reviews were excluded.

The search started with a selection of papers according to title, summary and key words by two reviewers acting in parallel and independently and including them in the study once the papers were entirely reviewed. We resolved disagreements on study selection and data extraction by consensus and discussion with other reviewers if needed. The references of all papers selected were then revised in search of other papers suitable to be included.

### 2.3. Data Analysis

A data-charting form was jointly developed by two reviewers to determine which variables to extract. The two reviewers independently charted the data and discussed the results. Two reviewers independently charted the data from each eligible article. Any disagreements were resolved through discussion between the two reviewers or further adjudication by a third reviewer.

Information collection of the documents was performed using a structured datasheet specifically developed by the authors following the JBI manual’s recommendations for data collection [14]. From each paper included, the following information was collected: (1) author and publication year. (2) title, (3) geographical location of the study, (4) design and aim of the study and (5) a summary of the results.

## 3. Results

**Figure 1:**
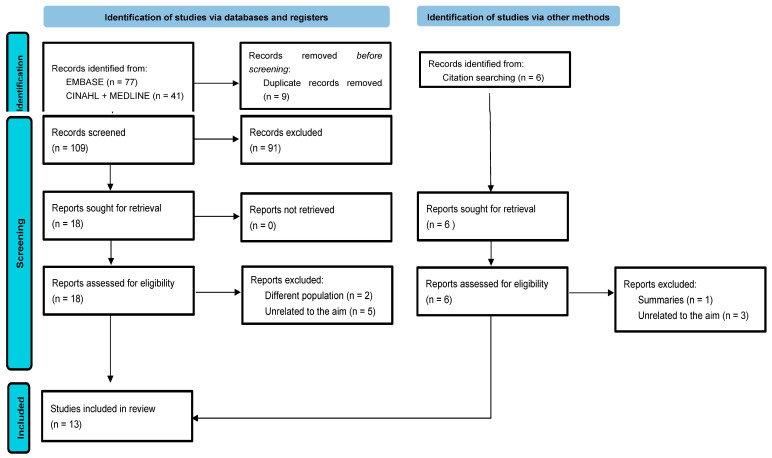
Flow diagram of the review process according to the PRISMA statement.

The initial search strategy identified a total of 118 studies in the database search, and duplicates were subsequently eliminated and selected according to the objective of this review, with a total of 11 studies included in this way. In addition, we were able to identify another six studies by other methods, of which two were selected after applying the exclusion criteria. Finally, a total of 13 studies were selected. The PRISMA flowchart is shown in Figure 1.

Table 2 shows the characteristics of all 13 studies included in this review. They were classified by author and publication year, context, design and aim, participants, the country where the included studies were carried out and the main findings. As for the study type, one was a systematic review [2], and eight were observational studies, including cross-sectional studies [11,15,16,17,18,19,20,21], prospective studies [10,22] and pre- and post-intervention studies [23,24].

## 4. Discussion

The literature on delayed effective discharge for non-medical reasons for patients admitted to acute hospitals in Spain is limited. 

### 4.1. Definition of the Term, Prevalence and Mean Admission Stays

To enable comparison over time between different studies a consistent definition of the concept of discharge delay, delayed discharge or bed blocking is needed. Considering that this is a subjective decision made by a physician, the question of when and why different patients are qualified as “bed blocking” should be carefully studied. Other expressions used as synonyms are long stays, discharge delay, transfer delay, inadequate hospitalization days, alternative level of care and barrier days [16].

The most common definition in the selected papers was that delayed discharge for non-clinical reasons or bed blocking is an extended period of stay starting when the patient is considered clinically stable for discharge but remains at the hospital for non-medical reasons [2].

Another term we found was inappropriate hospital use. This is defined as a hospital admission to provide care that could have been given in a less complex healthcare environment at a lower cost [23].

Two tools used to assess the need of admission and the causes of inadequacy were identified. On the one hand, there is the appropriateness evaluation protocol (AEP), which is best known and most frequently used and consists of explicit criteria on medical care, nursing care and patients’ clinical condition. Fulfillment of these criteria justifies the days of stay. Inversely, not meeting any criterion is considered an inadequate situation [15,21,22,23].

The other tool is adeQhos^®^. It is an easy-to-use, quick questionnaire filled in by the clinicians themselves, incorporating subjective clinical criteria [24].

The definition is also relevant to assess the selection of study subjects. Studies were found where the patients’ samples were patients who had already been given a discharge date by the hospital but were still occupying hospital beds for non-clinical reasons, namely patients deemed fit to be discharged by the admissions unit of the hospital but whose actual discharge was delayed for over 24 h [11,17,20].

Another study included patients who remained in the hospital despite having been declared clinically fit to be discharged by their attending physicians without specifying a minimum delay time [10]. 

Hence, there is no consensus as to when a case ought to be considered delayed discharge for non-clinical reasons. Although most authors agree that the patient remains in the hospital despite having been deemed clinically fit for discharge, there is a lack of objective criteria to define such fitness.

As for the prevalence data, the proportion was found to vary widely depending on whether the study focused on the total number of hospital beds or specific units, such as those of geriatric patients with a chronic disease. Thus, these findings should be taken with caution. The study by Pellico-López et al. during the period between 2007 and 2015 [11] found a prevalence of 0.93% for inadequate stays among the total number of discharges despite the University Hospital Marqués de Valdecilla (HUMV) being authorized to transfer patients to a private long-stay center, having an agreement with the national health service to provide functional recovery care and rehabilitation or palliative care as an intermediate care step between hospitalization and the community during the study period. This variation in prevalence data was also reflected in studies on other countries. For example, in the United Kingdom, they ranged between 1.6% and 60% [25].

The mean admission stays after being discharged varied widely between studies, ranging from 5 days (range: 3–12 days) [10] in a study only including internal medicine (IM) patients to 7.34 days in the studies carried out in the HUMV between 2007 and 2015 [11], with slight variations depending on the cause for admission, namely from 7.12 days in hip processes [16] to 7.61 days in admissions for pneumonia [17]. Data from three public hospitals in Cantabria from 2010 to 2014 revealed a mean delayed stay of 13.56 days [20].

### 4.2. Causes and Effects of Delayed Discharge

Causes of delayed discharge can be divided into clinical and care-related characteristics.

#### 4.2.1. Clinical Characteristics

As for the demographic characteristics, it was found that in the studies developed in the HUMV during the period from 2007 to 2015, the mean age of the patients was 77.28 years, and 65.97% of the patients were over 75 [11]. In stroke cases, the mean age increased to 79.7 years [18].

A previous study with IM patients showed a lower mean age of 75.74 years. Still, the mean age of patients with delayed discharge was higher than that of the total number of patients admitted to IM units [9].

Overall, the studies consulted agreed that patients over 75 have longer stays and are more likely to endure delayed discharges.

As far as sex, an even distribution was found.

Older age and diseases causing an abrupt functionality decline and dependency are the most common characteristics in frail elderly people. A study by Mendoza et al. found a higher prevalence of discharge delays in patients with acute cerebrovascular disease and problems related to the consumption of alcohol or benzodiazepines [10].

The complexity of patients’ processes has also been connected to delayed discharges, diagnosis-related groups (DRGs) used to measure it. The most frequent DRGs were those linked to stroke (6.1%) and other nervous system disorders (5.6%), pneumonia (4.1%) and hip procedures (3.3%) [11].

However, not all cases of delayed discharge were more complex. It was found that 10.0% of apparently simple DRGs had had delayed discharges, probably for having been admitted to a hospital due to overload or a lack of care and having to resort to hospitalization, pending social care [11]. This study provides data from various units, finding links between delayed discharges and units in charge and obtaining the longest stays in surgical units [11]. 

#### 4.2.2. Characteristics of Context of Care

The study by Mendoza et al. shows that in 98.8% of delayed discharge cases, the reason was some kind of socio-familial-related problem, whereas the remaining 1.17% were due to problems with the department in charge of patients’ transfers. The main reasons given for discharge delays were family overload, an inability to care for the patient due to being unable to balance the care needed by the patient and work life (51.8%) and lack of family or a social support network (21.8%) [10]. 

Family refusal to home-based care and lack of alternative care centers accounted for 6.2% of the cases each [23].

#### 4.2.3. Effects of Delayed Discharge

As for the effects of this problem, it was found that discharge delays for non-clinical reasons have negative repercussions aside from inefficient or inappropriate use of beds in acute hospitals. A review was found by Rojas-García et al. connecting delayed stays with an increased risk of healthcare-related infections, surgical intervention delays, death, loss of functional capacity and negative emotional impact on a patient presenting symptoms of anxiety and depression [2]. These findings are consistent with previous studies carried out in healthcare contexts similar to ours [1,26].

One of the causes analyzed in the selected studies was death. In the study by Mendoza et al., 15.7% of the patients whose discharge was delayed died pending the resource they had applied for due to nosocomial infection in 18% of the cases [10]. Lower numbers were obtained in the HUMV study, where 6.57% of delayed discharge cases died during the study period. It was found that the cases of delayed discharge admitted to medical units were more likely to die, and over a third were oncology patients [19].

### 4.3. Interventions

Despite the extent of the problem in Spain, only two papers analyzing interventions to deal with the issue were found.

A pre- and post-intervention study was carried out in 2007 in 10 acute care hospitals of Catalonia, including 2 intervention groups and their corresponding control groups. Intervention was performed using an adeQhos^®^ questionnaire in the patients’ visitor pass. The same reviewers evaluated adequacy using the AEP prior to and post intervention. No significant decrease in hospital inadequacy was observed after a low-intensity intervention [24].

A later study by Soria-Aledo et al. using the same protocol involved a retrospective audit of the first stage (pre) to analyze a random sample of medical records from patients admitted to a hospital using the AEP (control group). Then, an improvement cycle was performed, implementing measures to reduce admissions and inappropriate stays, and finally, a second evaluation was carried out, remeasuring admissions and inappropriate stays using a retrospective random sample of medical records (intervention group). The measures implemented were educational sessions with all clinical units to inform of the outcomes and discuss potential measures to reduce the inadequacy of admissions and stays, spread the use of the preliminary discharge report and create specific circuits to perform radiology studies on priority emergency patients and those newly diagnosed with cancer. Inadequate admissions decreased by 4.2% in the intervention group. Inappropriate stays also diminished significantly from 24.6% to 10.4%. Inadequate costs in the study sample dropped from EUR 147,044 to EUR 66,642 [23].

Various studies from countries with health and social contexts similar to those of Spain indicate that discharging people home or into community settings rapidly and safely after a stay in the hospital has long been a significant shared challenge for the health and care system, including Canada [1], USA [27] and England [28].

A study conducted by Romeyke and Stummer [29] in a German hospital found that complex therapies in hospital care in Germany lay the foundation for an integrative and interdisciplinary provision of services. They define the minimum criteria for the organization of a hospital, allow the integration of different therapeutic approaches and therefore lead to intensive and holistic treatment by a specially trained team. The German model can be considered a pilot model for the introduction of comprehensive patient-centered care in other hospitals around the world, which above all would lead patients with multiple pathologies to be reintegrated into the out-of-hospital environment.

This review comes with several limitations. Firstly, the literature available in Spain is scarce, and we do not have enough recent studies. Secondly, most works included are cross-sectional observational studies that do not allow causality inferences, and the samples lack representativity. Thirdly, not all authors used the same measuring tools because the outcomes may not be homogeneous. More research is needed on the topic to determine the reason for the problem of delayed discharge for non-clinical reasons. Moreover, the definition of the concept focuses only on acute hospital care for adults from the organization’s perspective. Aside from that, the identified studies are quantitative. No primary qualitative studies focusing on the personal experience of patients and families were found to allow a global overview of the problem. Furthermore, some of the studies did not provide sufficient information about the current status of the health system. Another limitation is that those studies that used the AEP did not take into account chronic patients with acute exacerbation of their diseases.

In conclusion, discharge delays are confirmed to be a multifactorial issue. They can be caused by factors internal or external to hospitals, as well as personal factors. The main reasons for the delay were similar among the studies found, and so were the medical characteristics of the patients, most of whom were elderly, frail and more dependent due to declining functional capacities. Little is mentioned about the socio-familial characteristics of the patients. Further research is thus needed on these variables to know the causes of the issue and potential interventions for improvement from the perspective of patients and families.

## Figures and Tables

**Table 1:** Search strategy used depending on database.

Database	Search Strategy	Search Date
EMBASE	((‘delayed discharges’ or ‘discharge delays’ or ‘bed blocking’ or ‘timely discharge’ or ‘unnecessary days’ or ‘inappropriate stays’) and (‘Spain’ or ‘Spanish’)).mp. (mp = title, abstract, full text, caption text)	28 March 2023
CINAHL	AB (delayed discharge or delayed discharge from hospital or bed-blocking or delayed transfer of care) AND TX (Spain or Spanish or España)	28 March 2023
MEDLINE	(AB delayed discharge OR AB delayed discharge from hospitals OR AB bed blocking OR delayed transfer of care) AND TX Spain OR TX Spanish OR TX España	28 March 2023

**Table 2:** Participants, design and main results of the studies included in the review.

Author and Year	Study Type	Aim	Participants and Context	Country	Main Findings
Pellico-López, Fernández-Feito et al. (2022) [18]	Descriptive, observational, cross-sectional and retrospective study	Describing the costs and characteristics of patients diagnosed with stroke and discharged late for non-clinical reasons and evaluating the connection between total stay duration and patient’s characteristics and care context	443 patients diagnosed with stroke and discharged late for non-clinical reasons in the HUMV (2007–2015)	Spain	Delayed discharges increase the total duration of stay by approximately a week. These patients with strokes have longer hospital stays, more complex care and higher costs than other cases of delayed discharges. Patients with strokes and delayed discharge are usually older patients, but when younger patients are affected, their length of stay is longer in relation to the sudden onset of the problem and the lack of adequate functional recovery resources or residential facilities for intermediate care. Patients who returned home after bed blocking had significantly higher lengths of total stay.
Pellico-López, Herrero-Montes, (2022) [19]	Descriptive, observational, cross-sectional and retrospective study	Describing the characteristics of patients deceased during delayed stays in terms of duration of hospital stay, patient’s characteristics and care context	198 patients deceased during their hospital stay after being discharged from the HUMV (2007–2015)	Spain	The study included all those patients identified as clinically fit for medical discharge by the hospital’s admission department but whose actual discharge was delayed by more than 24 h. Here, 6.57% of patients with delayed discharges for non-clinical reasons died during their hospital stays. The most common diagnosis among the deceased was simple pneumonia, likely caused by factors such as old age, comorbidity, fragility or complications arising from hospital infections.
Pellico-López, Fernández-Feito, Cantarero et al. (2021) [16]	Descriptive, observational, cross-sectional and retrospective study	Quantifying the connection between stay and its costs in hip processes with delayed discharge for non-clinical reasons	306 patients admitted to the HUMV (2007–2015) for hip processes with delayed discharges for non-clinical reasons	Spain	Average delayed stay was 7.12 days. The cost of delayed stay amounted to EUR 641,002.09. Up to 85.29% of patients lived in urban areas near the hospital, and 3.33% had been transferred to a long-stay center for recovery. The percentage of patients with hip procedures and delayed discharge was lower than in prior reports. However, their duration of stay was longer.
Pellico -López, Fernández-Feito et al. (2021) [17]	Descriptive, observational, cross-sectional and retrospective study	Understanding which characteristics are common in pneumonia patients compared with other cases of delayed discharge	170 patients diagnosed with pneumonia who were discharged late in the HUMV (2007–2015)	Spain	Pneumonia patients were older, less complex and had higher death rates than the rest of the patients.
Pellico -López et al. (2019) [11]	Descriptive, observational, cross-sectional and retrospective study	Quantifying the number of delayed discharge cases and inappropriate hospitalization days and identifying the use of health services linked to bed blocking	3015 patients with delayed discharges in the HUMV (2007–2015)	Spain	The characteristics most frequently associated with longer stays were the following: increased complexity, diagnosis implying lack of functional capacity, surgical treatment and having to wait for a destination when discharged or getting back home. Multiple-component interventions linked to discharge planning may favor inefficiency reduction minimizing unnecessary stays.
Pellico-López et al. (2018) [20]	Descriptive and retrospective study	Identifying which characteristics may influence the issue and quantifying inappropriate hospitalization days	This study included three public hospitals of a northern Spanish region (Cantabria) during 2010–2014	Spain	In the period from 2010 to 2014, 1415 bed-blocking cases were found in Cantabria hospitals waiting to be admitted to long-stay hospitals.
Rojas-García et al. (2018) [2]	Systematic review	Systematically reviewing delayed discharge experiences from the perspective of patients, health professionals and hospitals and their impact on patients’ outcomes and costs	37 papers were included, of which 2 were developed in Spain	OECD countries	Delayed discharge was associated with mortality, infections, depression and reductions in patients’ mobility and their daily activities. The qualitative studies highlighted the pressure to reduce discharge delays on staff stress and interprofessional relationships, with implications for patient care and well-being. Extra bed days could account for up to 30.7% of total costs. Most of the research was conducted poorly, which warrants precaution when considering its practical implications. The results suggest that the adverse effects of delayed discharges are both direct, due to the potential health problems they may cause in patients, and indirect, owing to increased pressure on healthcare workers.
Soria-Aledo et al. (2012) [23]	Descriptive, pre- and post-intervention and retrospective study	Reducing inappropriate admission and stays, as well as analyzing the hospital costs saved by inadequate stay reduction	1350 stays at J. M. Morales Meseguer Hospital	Spain	Inappropriate stays considerably decreased from 24.6% to 10.4%. Inadequacy cost in the study sample dropped from EUR 147,044 to EUR 66,642.
Mendoza Giraldo et al. (2012) [10]	Unicentric, observational, open and prospective study	Analyzing discharge delays for non-medical reasons at the IM unit of a third-level hospital and establishing the clinical and socio-familial factors linked to this situation	164 patients admitted to the IM unit of the HUVR whose discharges were delayed for non-medical reasons (between 1 February 2008 and 31 January 2009)	Spain	In total, 3.5% of discharges were delayed for non-medical reasons. Patients whose discharges were delayed were older and presented higher prevalence of acute cerebrovascular disease and problems related to alcohol or benzodiazepine consumption. The main reasons given for not being discharged were family overload or inability to provide care and lack of family or a social support network.
Soria-Aledo et al. (2009) [21]	Retrospective and descriptive study	Analyzing variables linked to inappropriate admissions and hospital stays and their economic repercussions	A total of 725 medical records and 1355 stays at J. M. Morales Meseguer Hospital were selected	Spain	The study found 7.4% of admissions and 24.6% of stays to be inappropriate. Most common causes of inappropriate stays were diagnosis or therapeutical procedures that could be performed on an outpatient basis, waiting for test results or consultations, a physician’s conservative attitude, bank holidays and lack of a diagnostic or treatment plan.
Monteis-Catot et al. (2007) [24]	Pre- and post-intervention study using «adeQhos^®^» questionnaire	Evaluating the impact of an intervention on the percentage of inappropriate stays (ISs) to verify the hypothesis that a simple information and participation intervention (adeQhos^®^) allows one to reduce the IS percentage	Design consisting of 2 intervention groups and their corresponding control groups in acute hospitals in Catalonia (708 patients per group)	Spain	Of all the stays reviewed (day before discharge), 41.1% were inappropriate. In hospitals with an intensity of intervention >60%, the proportion of IS decreased by 10.7 points in internal medicine and by 4.8 points in general surgery, while the proportion of IS increased in the control groups. No significant reduction in hospital inadequacy was observed after a low-intensity intervention.
Rodríguez-Vera, (2003) [15]	Observational and descriptive study	Determining admission and stay inadequacy at an IM unit using AEP-concurrent version	59 patients admitted to Juan Ramón Jiménez Hospital	Spain	Overall, 33% of stays were found to be inadequate. Waiting for complementary test results and inter consultations were the most common reasons for inadequate stays.
Zambrana-García et al. (2001) [22]	Observational, descriptive, and prospective study	Knowing the factors that may influence inadequate stays in an IM unit.	1046 of the 13,384 stays generated during 1998 in the IM unit of the Poniente Hospital.	Spain	A total of 176 stays were considered inadequate (16.8%). A logistic regression analysis revealed the main factors of stay inadequacy to be days of stay, day of the week and diagnosis on admission. Among the causes of inappropriate stays for non-medical reasons were medical treatment of the patient that was too conservative (37.5%), family refusal for home care (6.2%) and not having alternative care settings (6.2%).

HUMV = University Hospital Marqués de Valdecilla (by its Spanish acronym); AEP = appropriateness evaluation protocol; OECD Organisation for Economic Co-operation and Development; HUVR = Virgen del Rocío University Hospital (by its Spanish acronym); IM = internal medicine.

## Data Availability

Not applicable.

## Public Involvement Statement

There was no public involvement in any aspect of this research.

## Guidelines and Standards Statement

This manuscript was drafted against the Joanna Briggs Institute methodology for scoping review research.
